# Frequency and Systemic Associations of Focal Low-Trabeculation/Low-Density Intramedullary Jawbone Findings: A Retrospective CBCT Study

**DOI:** 10.3390/jcm15176636

**Published:** 2026-08-27

**Authors:** Marzena Dominiak, Wojciech Niemczyk, Artur Pitułaj, Witold Świenc, Sebastian Dominiak, Konstanty Sławecki, Łucja Janek, Jacek Matys

**Affiliations:** 1 Dental Surgery Department, Wroclaw Medical University, Krakowska 26, 50-425 Wroclaw, Poland; marzena.dominiak@umw.edu.pl (M.D.); artur.pitulaj@umw.edu.pl (A.P.); witold.swienc@umw.edu.pl (W.Ś.); konstanty.slawecki@umw.edu.pl (K.S.); 2 Medical Center of Innovation, Wroclaw Medical University, Krakowska 26, 50-425 Wroclaw, Poland; sebastian@dominiak.net.pl; 3 Statistical Analysis Centre, Wroclaw Medical University, Marcinkowskiego 2-6, 50-368 Wroclaw, Poland; lucja.janek@umw.edu.pl

**Keywords:** cone-beam computed tomography, intramedullary jawbone findings, trabecular rarefaction, fatty degenerative osteonecrosis of the jaw, marrow fatty infiltration, focal osteoporotic bone marrow defect, systemic diseases

## Abstract

**Background:** This exploratory study assessed the frequency of study-defined focal low-trabeculation/low-density intramedullary findings (FLDIFs) in a selected clinical cohort referred for cone-beam computed tomography (CBCT) and examined their associations with demographic, systemic, and radiographic variables. CBCT depicts mineralized trabecular architecture and relative attenuation but cannot directly demonstrate marrow fat or establish osteonecrosis; FDOJ/MFI were therefore considered only possible interpretations rather than imaging diagnoses. **Methods:** A single-center retrospective analysis included 1000 adults whose CBCT examinations met the study image-quality and anatomical-coverage requirements. FLDIF was defined as a descriptive radiological phenotype and was not considered a diagnosis of fatty degenerative osteonecrosis of the jaw (FDOJ), marrow fatty infiltration (MFI), or another specific disorder. Patients were classified as positive when at least one study-defined finding was present; one largest index finding per positive patient was used for finding-level analyses. **Results:** FLDIFs were identified in 452/1000 patients (45.2%; 95% CI: 42.1–48.3%) within this CBCT-referred cohort. Age was associated with finding presence in the multivariable model (OR 1.015 per year, 95% CI: 1.006–1.025; *p* = 0.001). Diabetes mellitus also showed an adjusted association (OR 1.647, 95% CI: 1.022–2.686; *p* = 0.042), which should be regarded as exploratory because of residual confounding and retrospective disease ascertainment. Panoramic radiographs were available for all 452 index findings; 180 (39.8%) were judged visible and 272 (60.2%) not visible. Visible index findings were larger (median 9.2 vs. 8.1 mm; pFDR = 0.003; |rank-biserial r| = 0.19), whereas the relative CBCT gray-scale comparison was not significant after FDR correction. Most index findings (412/452, 91.2%) were located in the mandibular retromolar regions. **Conclusions:** The 45.2% value represents the frequency of a study-defined, nonspecific radiological phenotype within a selected CBCT-referred cohort and should not be interpreted as population prevalence or as the prevalence of histopathologically confirmed FDOJ/MFI. The findings are hypothesis-generating; they do not establish a screening indication for CBCT based on age or diabetes. The biological and clinical significance of the imaging phenotype remains uncertain and requires external validation in independent cohorts and, when clinically indicated, histopathological or complementary imaging correlation.

## 1. Introduction

Focal areas of reduced cancellous trabeculation and relatively low radiographic density are encountered on dental CBCT, particularly in posterior mandibular marrow. Their interpretation is uncertain because similar appearances may reflect normal or age-related marrow variation, focal fatty replacement, focal osteoporotic bone marrow defect (FOBMD), or other local processes. The terminology used in the literature is therefore important. Fatty degenerative osteonecrosis of the jaw (FDOJ) refers to a proposed osteonecrotic/fatty-degenerative process, whereas marrow fatty infiltration (MFI) and bone marrow steatosis describe increased marrow adiposity and do not by themselves establish osteonecrosis. FOBMD is a distinct benign marrow alteration. Reduced trabeculation and low radiographic density on CBCT are nonspecific and cannot distinguish these entities without clinical and, when indicated, histopathological correlation [1,2,3,4,5,6,7]. In addition, CBCT gray-scale values are device- and protocol-dependent and should not be interpreted as standardized Hounsfield units or absolute bone-density measurements [8,9,10,11]. For these reasons, the present study uses the neutral term “focal low-trabeculation/low-density intramedullary finding” (FLDIF) for the measured radiological phenotype and discusses FDOJ, MFI, FOBMD, and physiological marrow conversion only as possible interpretations.

From an imaging-construct perspective, CBCT visualizes mineralized tissue architecture and provides device-dependent gray-scale information; it does not directly characterize marrow fat content and cannot establish osteonecrosis. Accordingly, FDOJ, MFI, bone marrow steatosis, and FOBMD are not treated as interchangeable labels for the primary endpoint in this study. They are considered only within the differential interpretation of the neutral FLDIF phenotype [1,2,3,4,6,7,8,9,10,11].

The clinical relevance of this phenotype is incompletely defined. Previous reports of FDOJ/FOBMD and marrow alterations have been dominated by histopathological series, case-based observations, and mechanistic or narrative literature, while large CBCT cohorts with patient-level systemic data remain limited [2,4,12,13,14,15,16]. Age is particularly relevant because mandibular marrow undergoes physiological fatty conversion over time [6,7]. Osteoporosis and other disorders affecting skeletal remodeling may plausibly influence trabecular architecture [17,18], while diabetes is associated with altered bone quality, microvascular dysfunction, and impaired remodeling [19,20,21]. Hypertension and cardiovascular disease were included as common systemic comorbidities with potential vascular and metabolic overlap, but the present analyses were exploratory and were not designed to establish causation [22]. A secondary radiographic question concerns whether CBCT-defined findings are also appreciable on panoramic radiographs, where superimposition and two-dimensional projection can obscure intramedullary changes [23,24,25,26,27,28].

The primary objective was to determine the frequency of study-defined FLDIFs within this selected CBCT-referred cohort and to explore their associations with age, sex, osteoporosis, arterial hypertension, cardiovascular disease, and diabetes mellitus. Secondary objectives were to describe the anatomical distribution and radiographic characteristics of the largest index finding per positive patient and to assess, in a targeted retrospective comparison, whether CBCT-defined index findings were also appreciable on available panoramic radiographs. The study was not designed to estimate prevalence in the general population, diagnose FDOJ/MFI histopathologically, evaluate diagnostic accuracy, or define indications for CBCT screening.

## 2. Materials and Methods

### 2.1. Study Design

This was a single-center exploratory retrospective observational study based on archived CBCT examinations and existing medical records from the Department of Oral Surgery, University Dental Center, Wroclaw Medical University, Wroclaw, Poland. The primary outcome was the presence or absence of the study-defined FLDIF radiological phenotype. Because no histopathological reference standard was available, this endpoint was treated exclusively as a descriptive imaging classification and not as a diagnosis of FDOJ, MFI, osteonecrosis, FOBMD, or any other specific pathological entity. The reported 45.2% therefore represents the frequency of this imaging phenotype within the analyzed CBCT-referred cohort rather than population prevalence. No CBCT examination was acquired specifically for this study; all imaging had been obtained previously as part of routine clinical care for reasons independent of the retrospective research protocol.

As this was a retrospective study based exclusively on previously acquired CBCT examinations and existing medical records, no additional diagnostic, therapeutic, surgical, or laboratory procedures were performed for the purpose of the study. Therefore, the study did not involve any direct physical risk to the participants. All clinical and radiographic data were analyzed anonymously, and personal data were processed in accordance with applicable data protection regulations and the principles of medical confidentiality. The study was conducted in accordance with the principles of the Declaration of Helsinki. The Bioethics Committee of Wroclaw Medical University, Wroclaw, Poland, confirmed that the study did not constitute a medical experiment and did not require a positive ethics committee approval under applicable Polish law. This confirmation was issued as Certificate No. 450/2025, reference number KBkanc 137/2025, dated 2 December 2025.

### 2.2. Study Population

The study population consisted of 1000 adult patients who underwent CBCT examination at the University Dental Center, Wroclaw Medical University, between 2019 and 2025 and met the eligibility criteria. The radiological review itself comprised exactly 1000 CBCT examinations. Examinations whose field of view did not extend sufficiently to include all required screening areas, including the mandibular retromolar regions, were not subjected to the study radiological assessment and were therefore not entered into the analytic cohort. The number of such non-reviewed examinations was not retained in the study dataset. The CBCT examinations had been obtained previously for routine clinical reasons independent of this retrospective study; the clinical indication for each examination was not extracted because indication was not a predefined study variable, and no CBCT was acquired specifically for research purposes. The patient was the primary unit of analysis. A patient was classified as FLDIF-positive if at least one study-defined focal finding was identified. When more than one suspicious finding was present, the finding with the largest diameter was selected as the index finding for finding-level measurements and analyses; thus, each positive patient contributed one index-finding record (*n* = 452). The index-finding strategy avoided multiple correlated measurements from the same patient in the primary finding-level analyses, but it preferentially selects larger findings and may influence the observed distributions of size, location, and panoramic visibility. The final analytic dataset contained one index-finding record per positive patient; additional non-index findings were not characterized in the final analysis. No a priori sample-size calculation was performed; the analysis used 1000 eligible reviewed records. With 452 positive outcomes and six prespecified patient-level predictors in the multivariable model, the overall number of outcome events was adequate for the fitted model, although sparse exposure categories—particularly osteoporosis (*n* = 20)—limit the precision of individual estimates.

The clinical indication for CBCT was not extracted as a predefined study variable, and no indication-stratified analysis can therefore be performed retrospectively from the analytic dataset. This absence is treated as a source of referral/selection bias rather than as evidence that the indication is unimportant: the cohort represents patients who had already undergone CBCT for routine clinical reasons, and the reported frequency must be interpreted within that selected setting.

The available retrospective analytic dataset contained one patient-level CBCT record per included patient. A separate source-level audit of repeat examinations across the complete imaging archive was not available and therefore is not reported (see Table 1).

### 2.3. Eligibility Criteria

#### 2.3.1. Inclusion Criteria

Patients were included if they met all of the following criteria:

Age ≥ 18 years at the time of CBCT examination;
Availability of a CBCT scan of sufficient diagnostic quality and anatomical coverage to evaluate the prespecified study screening areas, including the mandibular retromolar regions;
complete medical documentation containing information regarding the patient’s general health status.

#### 2.3.2. Exclusion Criteria

Patients were excluded if any of the following criteria were present:

Age below 18 years at the time of CBCT examination;
Insufficient CBCT image quality, including severe artifacts, image distortion, motion artifacts, or incomplete field of view;
incomplete medical documentation precluding analysis of clinical data.

### 2.4. CBCT Acquisition and Image Analysis

All analyzed examinations were performed using a Dentsply Sirona GALILEOS 3D system (Dentsply Sirona Inc., Charlotte, NC, USA). The recorded acquisition parameters were a focal spot size of 0.5 mm, tube voltage of 85 kV, tube current of 5–7 mA, and effective exposure time of 2–6 s. Because relative CBCT gray-scale values can vary with acquisition and reconstruction parameters, these measurements were used only for within-dataset exploratory comparisons and were not interpreted as Hounsfield units or absolute bone mineral density [8,10].

All examinations were acquired on the same CBCT system and assessed in the same software environment. Exact field-of-view, voxel, reconstruction, and software-version metadata were not retained as standardized analytic variables in the retrospective dataset. Eligibility therefore relied on direct confirmation that image quality and anatomical coverage were sufficient for the prespecified screening regions, including the mandibular retromolar areas.

CBCT examinations were retrospectively reviewed by a single examiner. The radiological screen was restricted to the prespecified anatomical regions listed below; accordingly, the reported patient-level frequency pertains to this region-based screening protocol rather than to every possible jawbone location. The selected regions reflected locations emphasized in previous descriptions of FDOJ/FOBMD and clinically relevant marrow alterations, particularly posterior mandibular and extraction-related sites [2,4,13,15].

For the radiological review, examinations were assessed only when the field of view adequately covered the required screening regions; scans without coverage extending to the mandibular retromolar areas were not classified and were not entered into the analytic cohort. The archived analytic table did not retain a separate maxilla-only/mandible-only/both-jaw FOV category or region-specific denominator. Consequently, the anatomical percentages reported below describe the distribution of selected index findings among included patients and should not be interpreted as region-specific prevalence estimates.

During CBCT classification, the examiner had access only to the patient ID displayed in the imaging system. This identifier was not linked within the review workflow to the patient’s demographic or medical data; therefore, the examiner was blinded to age, sex, diabetes, hypertension, other systemic diagnoses, and medical history during CBCT assessment. CBCT examinations were reviewed by one experienced examiner.

Observer reproducibility was evaluated using a randomly selected subset of 100 CBCT examinations (10% of the analyzed cohort). The primary examiner repeated the FLDIF classification and quantitative measurements after a 4-week interval. Intra-observer agreement for FLDIF presence/absence was Cohen’s kappa = 0.87. A second examiner, blinded to the first reading and to patient clinical data, independently evaluated the same subset; inter-observer agreement was kappa = 0.82. For quantitative measurements, intra-observer ICCs were 0.95 for largest index-finding diameter and 0.93 for the minimum relative CBCT gray-scale value, while inter-observer ICCs were 0.92 and 0.89, respectively. These results indicate good-to-excellent reproducibility of the study-defined classification and measurement procedure.

The study-defined FLDIF phenotype required the coexistence of two core radiological features within the same focal intramedullary area: (1) visibly reduced trabeculation/trabecular rarefaction and (2) lower relative radiographic density than the immediately surrounding cancellous bone. No universal numerical gray-scale threshold was used for classification. The category was deliberately descriptive: a positive FLDIF did not establish fatty infiltration, osteonecrosis, or another specific diagnosis. Findings with imaging features more consistent with an established alternative diagnosis—such as an odontogenic or residual cyst, a periapical inflammatory lesion, or a simple/traumatic bone cyst—were excluded from the study-defined phenotype rather than being retained merely because they were radiolucent. The transition from the focal abnormality to adjacent cancellous bone was used for visual delineation during measurement.

To make the study endpoint more operational and reproducible, the classification logic is reported as the following checklist: (1) verify adequate image quality and field-of-view coverage of the screened region; (2) identify a focal intramedullary area rather than relying on diffuse background cancellous variation; (3) require both visibly reduced trabeculation/trabecular rarefaction and lower relative radiographic density than immediately adjacent cancellous bone; (4) do not assign FLDIF when the imaging pattern is better explained by a specific established entity such as a periapical inflammatory lesion, odontogenic/residual cyst, or simple/traumatic bone cyst; (5) treat relationships with previous extraction sites, implants, and adjacent teeth as contextual features rather than diagnostic proof of FDOJ/MFI; and (6) perform diameter and relative gray-scale measurements only after the focal phenotype has been identified. No numerical gray-scale threshold was used. This checklist operationalizes the descriptive imaging phenotype; it is not a validated diagnostic algorithm for FDOJ, MFI, FOBMD, or osteonecrosis.

Candidate findings were assessed across multiplanar reconstructions to confirm that the low-trabeculation/low-density appearance represented a focal intramedullary finding rather than an isolated single-view artefact. No universal numerical gray-scale threshold was used.

The prespecified screening regions were:

Mandibular retromolar regions, including areas distal to the third molar region;
Mandibular premolar regions;
Maxillary tuberosity regions;
Maxillary interincisal region;
Mandibular interincisal region;
Peri-implant regions, when present.

Morphology, trabecular pattern, relative radiographic density, anatomical location, and relationship to adjacent teeth/implants or previous treatment sites were considered as contextual imaging features; however, local context was not used to assign a specific pathological diagnosis. The phenotype was not assigned when a better-defined alternative radiographic diagnosis was considered more plausible, thereby avoiding conflation of nonspecific marrow changes with established cystic or inflammatory jaw lesions.

For each index FLDIF, the following parameters were included in the analytic dataset:

index-finding location;
largest index-finding diameter;
minimum relative CBCT gray-scale value within the index finding;
presence of previous tooth extraction in the index-finding area, when documented in the archived record; extraction history was analyzed descriptively only;
implant-related distance was recorded when applicable but was not included in the present inferential analyses;
visibility or non-visibility of the index finding on panoramic radiography.

Relative CBCT gray-scale values were used only as within-device, within-dataset descriptors. They were not interpreted as Hounsfield units, absolute density, or values transferable across devices/protocols [8,10]. The minimum value was retained because it was the descriptor specified in the original data collection; it should be interpreted cautiously because a minimum is more sensitive than a mean/median ROI value to local noise or artefact.

The largest index-finding diameter and the minimum relative CBCT gray-scale value were recorded in the same software environment used for image review. The largest diameter represented the greatest visually defined extent of the index finding. The minimum relative gray-scale value was retained as the device-specific descriptor specified in the original data collection. A fully standardized retrospective ROI-placement protocol was not retained in the archived study documentation; consequently, these quantitative gray-scale measurements should be interpreted as exploratory within-dataset descriptors rather than standardized density measurements.

### 2.5. Panoramic Radiography Assessment

For every CBCT–panoramic pair included in the comparative analysis, both examinations had been obtained during the same diagnostic visit. In the routine workflow of the center, panoramic radiography is generally used as the first-line radiographic examination, while CBCT is added when the clinical situation requires three-dimensional imaging. No CBCT was acquired specifically for research purposes. If panoramic radiography and CBCT had been performed at a greater temporal interval rather than during the same visit, that pair was not included in the comparative analysis. Accordingly, a suitable same-visit panoramic radiograph was available for each of the 452 FLDIF-positive patients included in the index-finding comparison (180 visible and 272 not visible). The panoramic assessment was a paired retrospective comparison of whether a CBCT-defined index finding was also appreciable on two-dimensional imaging; it was not designed as an independent diagnostic-accuracy study and had no histopathological reference standard. Index findings were classified as visible or not visible on panoramic radiography, and visibility was compared with index-finding size and relative CBCT gray-scale values.

The retrospective source documentation did not retain a standardized variable documenting whether panoramic interpretation was fully independent of the CBCT assessment. Accordingly, potential observer/incorporation bias cannot be excluded and is acknowledged as a limitation of the panoramic comparison.

Only same-visit CBCT–panoramic pairs of sufficient diagnostic quality were included in the comparative analysis. Detailed panoramic acquisition parameters were not included as standardized variables in the analytic dataset.

### 2.6. Clinical Data

Patient-level variables available consistently for the planned analyses were age, sex, osteoporosis, arterial hypertension, cardiovascular disease, and diabetes mellitus.

Disease status was based on diagnoses recorded in the available medical documentation. The retrospective dataset did not separately encode the provenance of each entry (for example, physician-confirmed diagnosis versus patient-reported history), and cardiovascular disease was available only as a composite source variable rather than as individual cardiovascular diagnoses. Diabetes type, duration, treatment, and metabolic control were not available as standardized variables. These limitations were considered when interpreting the systemic-disease analyses. The multivariable model was exploratory and included all six patient-level variables listed above; no missing values were present for these variables in the 1000 analyzed records.

The analytic dataset did not contain standardized patient-level covariates for smoking, body mass index, antiresorptive therapy, systemic corticosteroid exposure, oncological treatment, periodontal status, number of missing teeth, or detailed medication profiles affecting bone metabolism. Previous extraction and implant relationships had been recorded locally for index findings but were not available as complete patient-level covariates for multivariable adjustment. These unmeasured or incompletely standardized factors may confound the observed age- and diabetes-related associations.

### 2.7. Statistical Analysis

Statistical analyses were performed in R version 4.5.3. The analyses used base functions from the stats package together with readxl and car 1.5.0. where applicable. Supplementary Table S3 summarizes the principal statistical functions and model specification used in the analysis.

The primary descriptive measure was the frequency (proportion) of FLDIF-positive patients within the selected CBCT-referred cohort, reported with a 95% confidence interval. Age, sex, osteoporosis, arterial hypertension, cardiovascular disease, and diabetes mellitus were compared between FLDIF-positive and FLDIF-negative patients. A Cochran–Armitage trend test was additionally calculated from the ordered age-group counts in Table 2. Finding-level analyses were restricted to the single largest index finding from each positive patient.

Continuous variables were assessed using Q–Q plots and, because distributions were non-normal, are presented as median and interquartile range (Me [Q1–Q3]) and compared with the Mann–Whitney U test. For the panoramic-visibility comparisons, absolute rank-biserial correlations were calculated from the reported Mann–Whitney statistic to provide a nonparametric effect-size estimate.

Categorical variables were analyzed with Pearson’s chi-square test when expected-cell assumptions were adequate; Fisher’s exact test was used for osteoporosis because of the small exposed subgroup (*n* = 20). Unadjusted odds ratios with 95% confidence intervals were calculated for the principal binary comparisons. Benjamini–Hochberg false discovery rate (FDR) correction was applied as one prespecified exploratory family to the eight univariate *p*-values presented in Table 3: age, sex, osteoporosis, arterial hypertension, cardiovascular disease, diabetes mellitus, panoramic visibility versus relative gray-scale value, and panoramic visibility versus index-finding size. A single family was retained because these comparisons were interpreted jointly as exploratory analyses of the same study-defined phenotype.

An exploratory multivariable logistic regression model included age, sex, osteoporosis, arterial hypertension, cardiovascular disease, and diabetes mellitus simultaneously (*n* = 1000 complete cases). Results are reported as odds ratios (ORs) with 95% confidence intervals. Variance inflation factors were used to assess multicollinearity and Cook’s distance (reference value 4/n) was inspected for potentially influential observations. Age was modeled as a continuous term on the logit scale. Functional form was assessed using an age × log(age) term and a restricted cubic-spline comparison; neither analysis provided evidence of clinically relevant non-linearity (age × log(age), *p* = 0.41; spline-versus-linear likelihood-ratio comparison, *p* = 0.37), supporting retention of the linear age term.

Seven observations exceeded the 4/n Cook’s-distance reference value. After excluding these observations, the age estimate remained materially unchanged (OR 1.015 per year, 95% CI 1.005–1.026; *p* = 0.004). The diabetes estimate was similar in magnitude but became borderline/non-significant (OR 1.59, 95% CI 0.98–2.59; *p* = 0.061), reinforcing the exploratory interpretation of this association. The full model was significant versus the intercept-only model (likelihood-ratio χ^2^ = 27.6, df = 6, *p* < 0.001), with AIC = 1368.2, Nagelkerke R^2^ = 0.041, and Hosmer–Lemeshow χ^2^ = 7.1 (df = 8, *p* = 0.53). A two-sided *p*-value < 0.05 was considered statistically significant, with FDR-adjusted *p*-values used for the exploratory univariate family described above.

## 3. Results

### 3.1. Frequency of Study-Defined Findings and Age-Related Differences

**Figure 1:**
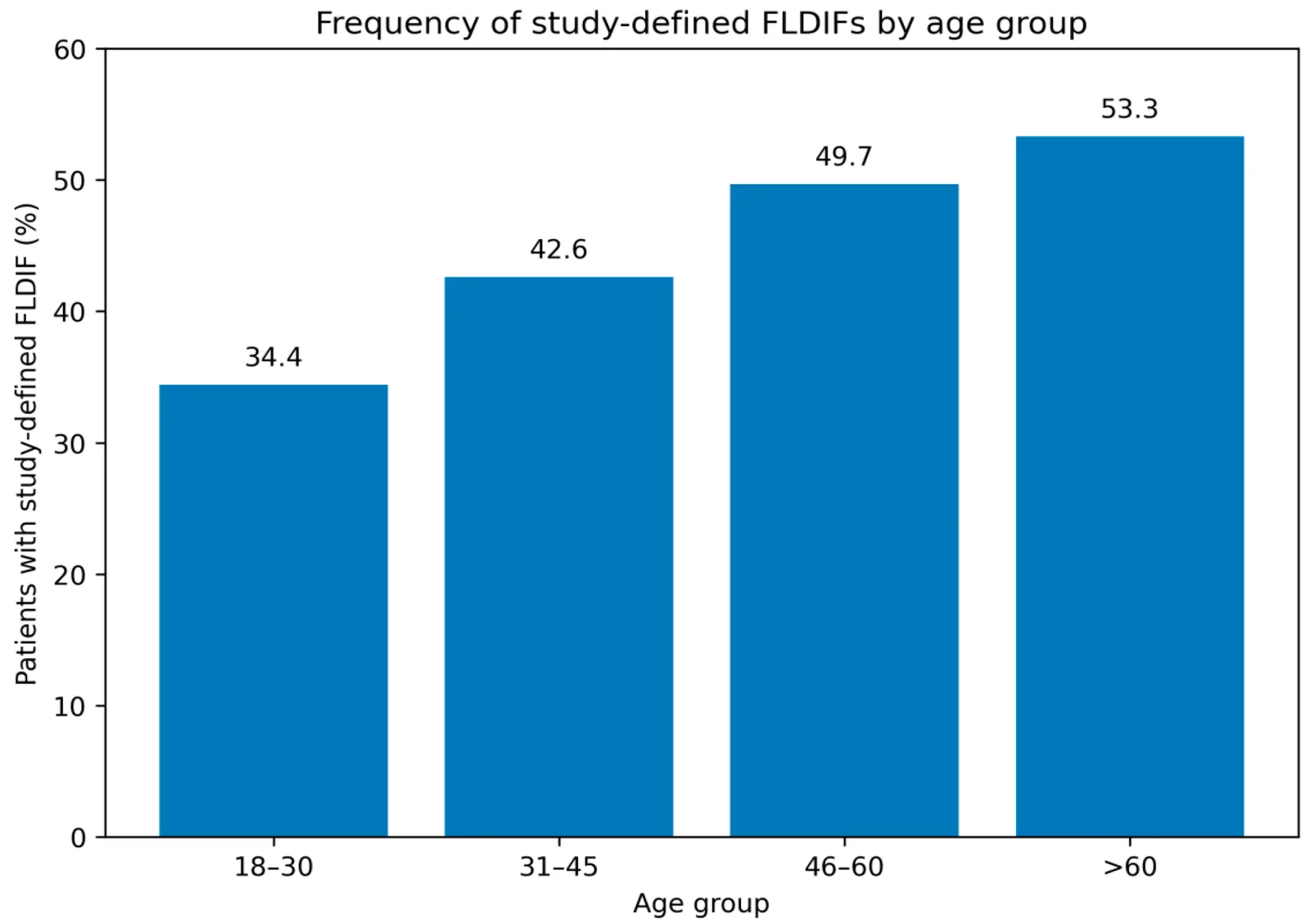
Frequency of study-defined FLDIFs according to age group in the analyzed CBCT-referred cohort.

Among the 1000 patients in this selected CBCT-referred cohort, 452 were classified as having at least one study-defined FLDIF, corresponding to a cohort frequency of 45.2% (95% CI: 42.1–48.3%) (Table 2). This proportion is not an estimate of prevalence in the general adult population. The observed proportions increased across ordered age groups from 34.4% at 18–30 years to 53.3% at >60 years; a Cochran–Armitage trend test based on Table 2 counts supported an ordered increase (Z = 4.35, *p* < 0.001) (Table 2, Figure 1). FLDIF-positive patients were older than FLDIF-negative patients (Me 49 [35–57] vs. 45.5 [29–52] years, *p* < 0.001; pFDR < 0.001) (Table 3).

### 3.2. Associations with Sex and Systemic Diseases

No statistically significant univariate association was observed for sex, cardiovascular disease, or osteoporosis (Table 2 and Table 3). The osteoporosis subgroup was small (*n* = 20); Fisher’s exact test yielded *p* = 0.659, and the corresponding unadjusted OR was 1.22 (95% CI: 0.50–2.95), indicating substantial imprecision. Arterial hypertension was associated with FLDIF presence in the univariate analysis (53.1% vs. 43.1%; *p* = 0.012; pFDR = 0.024; unadjusted OR 1.49, 95% CI: 1.10–2.03). Diabetes mellitus was also associated in the univariate analysis (60.3% vs. 43.9%; *p* = 0.008; pFDR = 0.021; unadjusted OR 1.94, 95% CI: 1.21–3.10). These estimates are descriptive/exploratory and do not account for unmeasured confounding.

### 3.3. Index-Finding Location

The largest index findings were strongly concentrated in the mandibular retromolar regions: 209/452 (46.2%) were on the left and 203/452 (44.9%) on the right, together accounting for 412/452 (91.2%) of index findings (Table 4). In Table 4, the retromolar categories are described anatomically as the regions distal to teeth 38 and 48. Table 4 describes only the location of the single largest index finding per positive patient and must not be interpreted as the distribution of all findings in the cohort. Additional non-index findings were not characterized in the final analytic dataset.

Among the 412 retromolar index findings, 247 (60.0%) were located at sites with a documented previous molar or third-molar extraction, whereas 165 (40.0%) had no previous molar or third-molar extraction recorded at the index-finding site.

### 3.4. Index-Finding Characteristics and Panoramic Visibility

Among the 452 FLDIF-positive patients, the median largest index-finding diameter was 8.5 mm [7.1–9.8] and the median minimum relative CBCT gray-scale value was −424.5 [−502.2 to −359.0] (Table 5). A suitable panoramic radiograph was available for all 452 index findings: 180 (39.8%) were judged visible and 272 (60.2%) not visible. This was a targeted retrospective comparison of CBCT-defined sites and not a diagnostic-accuracy assessment.

**Figure 2:**
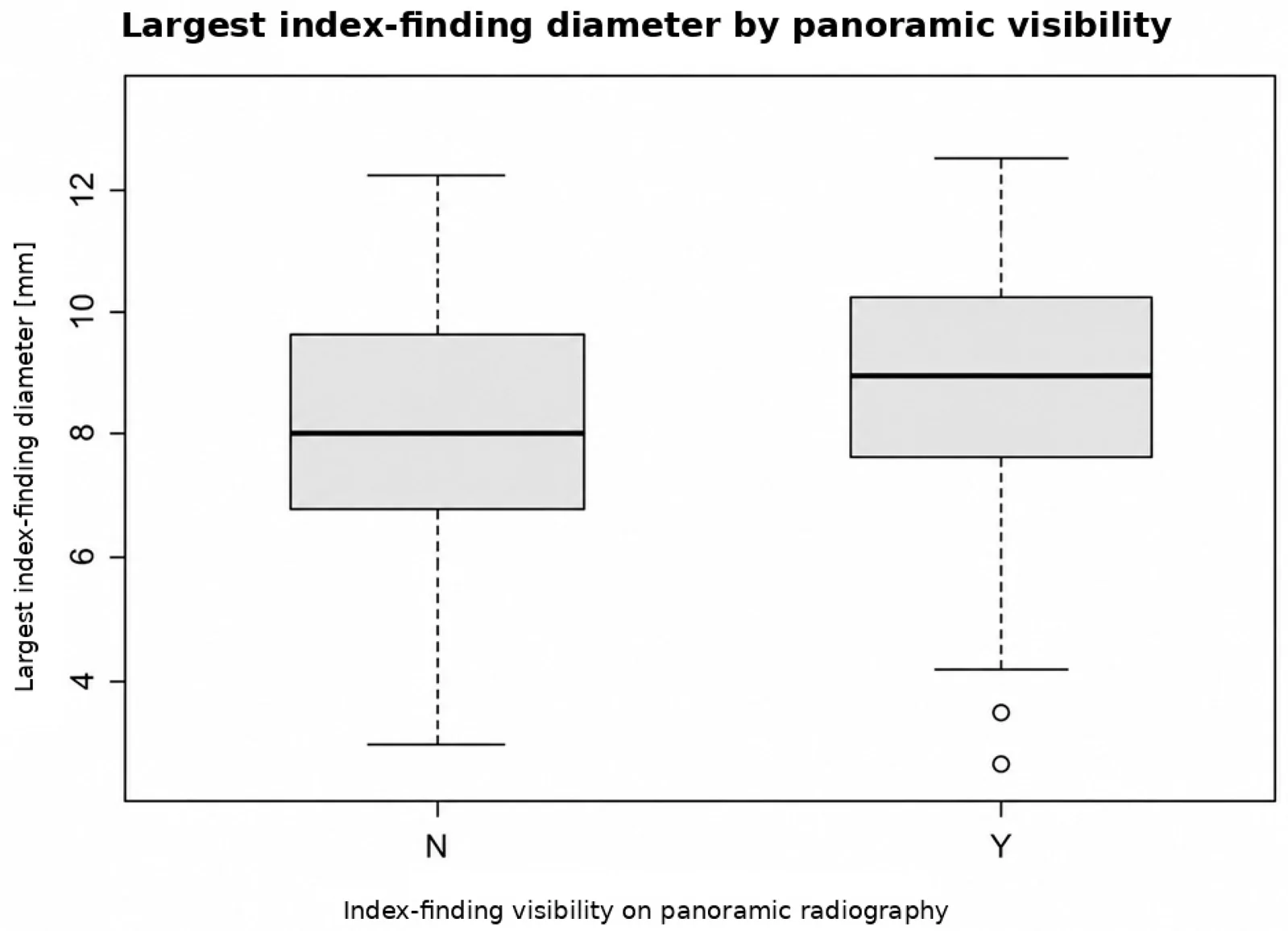
Largest index-finding diameter according to visibility on panoramic radiography.

**Figure 3:**
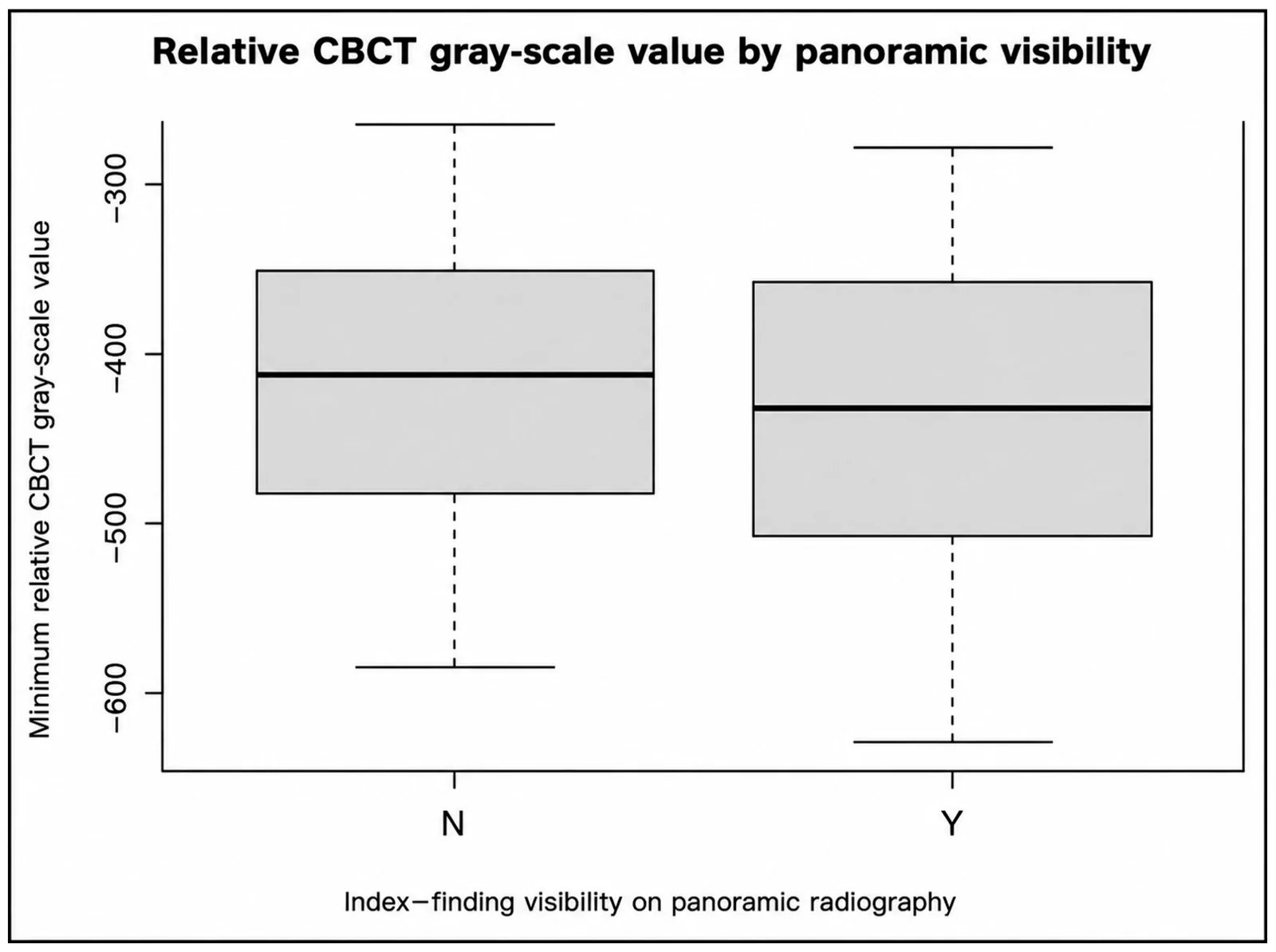
Relative CBCT gray-scale values of index findings according to visibility on panoramic radiography.

Index findings judged visible on panoramic radiography were larger than those judged not visible (Me 9.2 [7.5–10.1] vs. 8.1 [6.9–9.7] mm; *p* < 0.001; pFDR = 0.003; |rank-biserial r| = 0.19) (Table 3, Figure 2). Relative CBCT gray-scale values did not differ statistically after FDR correction (Me −437.5 [−517.2 to −362.5] vs. −418.0 [−494.0 to −355.8]; *p* = 0.050; pFDR = 0.080; |rank-biserial r| = 0.11) (Table 3, Figure 3).

### 3.5. Exploratory Multivariable Logistic Regression

In the exploratory multivariable logistic regression (*n* = 1000 complete cases), age and diabetes mellitus were statistically associated with FLDIF presence after simultaneous adjustment for sex, osteoporosis, arterial hypertension, cardiovascular disease, and diabetes (Table 6). The age OR was 1.015 per year (95% CI: 1.006–1.025; *p* = 0.001), corresponding to an OR of 1.161 per 10-year increase (95% CI: 1.062–1.280) under the model’s linear-logit assumption. Diabetes showed an adjusted OR of 1.647 (95% CI: 1.022–2.686; *p* = 0.042). The latter estimate is modest, has a lower confidence limit close to 1, and is potentially sensitive to residual confounding and influential observations; it should therefore be considered hypothesis-generating rather than evidence of a specific causal systemic–jawbone relationship. Hypertension was not statistically significant after adjustment, and the osteoporosis estimate was imprecise because only 20 patients had osteoporosis.

## 4. Discussion

This retrospective study found that 452 of 1000 patients in a selected university dental-center CBCT cohort met the study-defined FLDIF criteria. This 45.2% proportion should be interpreted only within the examined CBCT population and the prespecified region-based screening protocol. It is not a population prevalence estimate and does not indicate that 45.2% of adults have FDOJ, MFI, osteonecrosis, or another histopathologically defined disease. All CBCT examinations had been acquired for routine clinical reasons independent of the study, and none was performed specifically for research. The clinical indication for individual CBCT examinations was not extracted because it was outside the predefined study variables; therefore, indication-specific selection effects cannot be quantified. In addition, examinations without sufficient anatomical coverage, including coverage extending to the required retromolar regions, were not subjected to the study radiological assessment. These features reinforce that the observed frequency is cohort-specific and should not be generalized to the general population.

The lack of indication-level CBCT data further limits interpretation of the cohort frequency. Implant planning, impacted teeth, surgical assessment, endodontic questions, or other referral reasons may differ in their age distribution, extraction history, tooth-loss burden, and local osseous context. Because these indications were not encoded as analytic variables, their contribution cannot be separated from the observed radiological phenotype. Likewise, the absence of region-specific FOV denominators precludes interpreting Table 4 as a prevalence map of individual jaw regions.

Age showed the most consistent patient-level association. However, the biological interpretation is not specific. Mandibular marrow normally undergoes age-related conversion toward a more fatty composition, and marrow adiposity increases with aging [6,7,29,30]. Because 91.2% of the selected index findings were retromolar and the imaging phenotype itself was defined by reduced trabeculation and lower relative density, physiological marrow conversion or age-related cancellous variation is a plausible competing explanation for at least part of the age association. The present data did not measure perfusion, marrow adipocyte content, osteonecrosis, or histological remodeling; therefore, mechanistic explanations remain hypotheses rather than demonstrated pathways. Additional nonlinear-age analyses did not indicate a material departure from linearity (age × log(age), *p* = 0.41; spline-versus-linear comparison, *p* = 0.37).

Diabetes mellitus showed an adjusted association with FLDIF presence (OR 1.647, 95% CI: 1.022–2.686), but this result requires proportionate interpretation. Diabetes can affect bone quality through mechanisms that include altered remodeling, chronic inflammation, and microvascular dysfunction [19,20,21], yet none of these mechanisms was measured here. Diabetes type, duration, glycemic control, medication exposure, smoking, body mass index, periodontal status, tooth loss, corticosteroid or antiresorptive use, and several local dental factors were not available as standardized covariates. The confidence interval is relatively wide and its lower bound is close to 1; consequently, the association is exploratory and susceptible to residual confounding. It does not establish diabetes as an indication for CBCT. Hypertension was associated only in univariate analysis and was attenuated after adjustment, while cardiovascular disease showed no statistically significant association. Osteoporosis was present in only 20 patients; its wide confidence interval means that a non-significant result should not be interpreted as evidence of no association.

In particular, the adjusted diabetes estimate should be regarded as hypothesis-generating. The model cannot distinguish a direct diabetes-related marrow effect from confounding by smoking, obesity, periodontal disease, tooth loss, previous extraction, medications affecting bone metabolism, or other systemic/local factors that were unavailable as standardized covariates. The result therefore supports further investigation but not a disease-specific imaging recommendation.

The panoramic comparison should likewise be interpreted narrowly. All 452 selected index findings had a same-visit panoramic radiograph; CBCT was added in routine care when three-dimensional imaging was clinically required, and image pairs obtained at longer intervals were not included in the comparison. No independent clinical or histopathological reference standard was used, and the retrospective source documentation did not allow full verification of reader independence from the CBCT assessment. Potential observer/incorporation bias therefore cannot be excluded. This is not a sensitivity/specificity study and does not establish diagnostic superiority of CBCT or diagnostic inadequacy of panoramic imaging. The directly supported observation is that larger CBCT-defined index findings were more often appreciable on panoramic images, with a small rank-biserial effect size (|r| = 0.19), whereas relative gray-scale values were not significantly different after FDR correction. These results do not justify CBCT screening in older or diabetic patients; CBCT should continue to be obtained only for appropriate clinical indications.

These data should not be used to lower the clinical threshold for CBCT acquisition. Under radiation-justification principles, CBCT remains an adjunctive three-dimensional examination that should be obtained only when clinically indicated by the patient’s diagnostic problem; neither older age nor diabetes alone is established here as an indication. The present study also does not show that recognition of an FLDIF changes diagnosis, treatment, prognosis, or patient outcome.

The marked posterior mandibular concentration deserves particular caution. Previous FOBMD reports commonly describe mandibular molar or edentulous sites [2,13,15], but FOBMD is not synonymous with the present CBCT phenotype. Among the 412 retromolar index findings, 247 (60.0%) were located at sites with a documented previous molar or third-molar extraction, while 165 (40.0%) had no such extraction recorded at the index-finding site. This descriptive association cannot establish that extraction caused the imaging phenotype, because extraction history was not available as a complete patient-level covariate for multivariable adjustment and the interval between extraction and CBCT was not analyzed. In addition, selecting only the largest finding per positive patient may overrepresent locations where larger findings occur and can increase the probability of panoramic visibility. Differential interpretations of a FLDIF include physiological marrow variation, focal fatty replacement/MFI, FOBMD, FDOJ as proposed in the literature, and other local marrow changes. Established cystic or inflammatory lesions remain separate diagnostic entities; because imaging overlap can occur, definitive pathological labeling requires appropriate clinical and, when indicated, histopathological correlation [4,12,15,16,31,32].

Several limitations materially constrain interpretation. First, this was a single-center retrospective study of a clinically selected CBCT population. Exactly 1000 examinations meeting the required image-quality and anatomical-coverage criteria were radiologically reviewed; examinations without adequate coverage were not assessed, and the number of such non-reviewed archive examinations was not retained. Individual clinical indications for CBCT were not extracted, so indication-specific selection effects cannot be quantified. Second, the primary outcome was a partly subjective imaging phenotype. The examiner was blinded to demographic and systemic medical information because only an unlinked patient ID was available during CBCT assessment. Reproducibility was assessed in a 100-scan subset and showed good-to-excellent agreement (intra-/inter-observer kappa 0.87/0.82; ICC range 0.89–0.95). Nevertheless, external reproducibility of the FLDIF classification remains to be established in independent cohorts and with additional readers. Third, the screening was restricted to prespecified anatomical regions and finding-level analyses used only the largest index finding, so additional findings and the full finding distribution were not represented. Fourth, there was no histopathological reference standard and no prospective follow-up. Fifth, relative CBCT gray-scale values were device/protocol specific, and the retrospective documentation did not retain a fully standardized ROI-placement protocol. Sixth, the CBCT–panoramic comparison was restricted to same-visit image pairs, but reader independence could not be fully established retrospectively, so observer/incorporation bias cannot be excluded. Seventh, systemic diseases were derived retrospectively from medical records; provenance and disease severity were not standardized, and several potential systemic and local confounders were unavailable. Eighth, the exploratory logistic model had modest explanatory power (Nagelkerke R^2^ = 0.041), and the diabetes estimate became non-significant at the conventional 0.05 threshold after exclusion of seven observations above the 4/n Cook’s-distance reference. Finally, the osteoporosis subgroup was very small, limiting precision.

The principal endpoint remains an imaging-defined phenotype rather than a validated disease entity. CBCT cannot directly identify marrow fat or osteonecrosis, indication-specific referral bias cannot be quantified, region-specific denominators were not retained, and several major systemic and local confounders were unavailable. These constraints limit the external validity and etiological interpretation of the observed 45.2% cohort frequency.

Taken together, the results should be viewed as hypothesis-generating radiological observations. They support further work on reproducible imaging criteria and on the relationship between local marrow appearance, age, dental/extraction history, and systemic disease, but they do not establish the pathological nature of the FLDIF phenotype or demonstrate that identifying it changes patient management or outcomes.

## 5. Conclusions

Study-defined focal low-trabeculation/low-density intramedullary findings were identified in 45.2% of this selected CBCT-referred cohort. This value is cohort-specific and should not be generalized as population prevalence or interpreted as the prevalence of histopathologically confirmed FDOJ/MFI. Older age was associated with the imaging phenotype, but physiological age-related marrow conversion is an important alternative explanation. Diabetes mellitus showed a modest adjusted association (OR 1.647, 95% CI: 1.022–2.686) that remains exploratory because of retrospective disease ascertainment, residual confounding, and sensitivity concerns. Among the largest index findings, 91.2% were located in mandibular retromolar regions, and larger index findings were more often appreciable on panoramic radiographs. The study does not establish diagnostic accuracy, clinical benefit, or a screening indication for CBCT based on age or diabetes. Prospective studies using explicit operational criteria, independent multi-reader reproducibility assessment, complete local dental/extraction data, and clinical/histopathological or complementary imaging correlation are needed to determine the pathological and clinical significance of this radiological phenotype.

Accordingly, the study identifies a radiological phenotype rather than a histologically or MRI-validated disease entity. The association with age may partly reflect physiological age-related mandibular marrow conversion, and the biological and clinical significance of the high cohort frequency remains uncertain.

## Figures and Tables

**Table 1:** Descriptive characteristics of the analyzed CBCT-referred cohort (*n* = 1000).

Characteristic	Overall	Sex-Stratified Information
Age, years	Median [Q1–Q3]: 47 [31–54]	—
Sex	Female: 526 (52.6%); Male: 474 (47.4%)	—
Osteoporosis	Yes: 20 (2.0%); No: 980 (98.0%)	Female: 13/526 (2.5%); Male: 7/474 (1.5%)
Arterial hypertension	Yes: 211 (21.1%); No: 789 (78.9%)	Female: 118/526 (22.4%); Male: 93/474 (19.6%)
Cardiovascular disease	Yes: 88 (8.8%); No: 912 (91.2%)	Female: 40/526 (7.6%); Male: 48/474 (10.1%)
Diabetes mellitus	Yes: 78 (7.8%); No: 922 (92.2%)	Female: 39/526 (7.4%); Male: 39/474 (8.2%)
Study-defined FLDIF present	Yes: 452 (45.2%); No: 548 (54.8%)	Female: 242/526 (46.0%); Male: 210/474 (44.3%)

Q1–Q3—interquartile range; FLDIF—focal low-trabeculation/low-density intramedullary finding.

**Table 2:** Frequency of study-defined FLDIFs within the analyzed CBCT-referred cohort.

Variable	FLDIF Present, *n* (%)	FLDIF Absent, *n* (%)
Study-defined FLDIF	452 (45.2%) 95% CI: 42.1–48.3%	548 (54.8%) 95% CI: 51.7–57.9%
Sex		
Female	242 (46.0%)	284 (54.0%)
Male	210 (44.3%)	264 (55.7%)
Age groups		
18–30 years	83 (34.4%)	158 (65.6%)
31–45 years	86 (42.6%)	116 (57.4%)
46–60 years	194 (49.7%)	196 (50.3%)
>60 years	89 (53.3%)	78 (46.7%)
Osteoporosis		
Yes	10 (50.0%)	10 (50.0%)
No	442 (45.1%)	538 (54.9%)
Arterial hypertension		
Yes	112 (53.1%)	99 (46.9%)
No	340 (43.1%)	449 (56.9%)
Cardiovascular disease		
Yes	46 (52.3%)	42 (47.7%)
No	406 (44.5%)	506 (55.5%)
Diabetes mellitus		
Yes	47 (60.3%)	31 (39.7%)
No	405 (43.9%)	517 (56.1%)

**Table 3:** Exploratory univariate comparisons for the study-defined FLDIF phenotype and index-finding characteristics.

Comparison	Groups Compared	Results in Groups	Test Statistic	df	*p*-Value	FDR-Adjusted *p*-Value
Age of FLDIF-positive vs. FLDIF-negative patients	Y: 452 N: 548	Me [Q1–Q3]: 49 [35–57] Me [Q1–Q3]: 45.5 [29–52]	W = 103,476	—	<0.001	<0.001
Sex	Female: 526 Male: 474	242/526 (46.0%) 210/474 (44.3%)	χ^2^ = 0.227	1	0.633	0.659
Osteoporosis	Y: 20 N: 980	10/20 (50.0%) 442/980 (45.1%)	Fisher’s exact	—	0.659	0.659
Arterial hypertension	Y: 211 N: 789	112/211 (53.1%) 340/789 (43.1%)	χ^2^ = 6.308	1	0.012	0.024
Cardiovascular disease	Y: 88 N: 912	46/88 (52.3%) 406/912 (44.5%)	χ^2^ = 1.648	1	0.199	0.266
Diabetes mellitus	Y: 78 N: 922	47/78 (60.3%) 405/922 (43.9%)	χ^2^ = 7.097	1	0.008	0.021
Index-finding visibility on panoramic radiography vs. relative CBCT gray-scale value	Y: 180 N: 272	Me [Q1–Q3]: −437.5 [−517.2 to −362.5] Me [Q1–Q3]: −418.0 [−494.0 to −355.8]	W = 27,149	—	0.050	0.080
Index-finding visibility on panoramic radiography vs. index-finding size	Y: 180 N: 272	Me [Q1–Q3]: 9.2 [7.5–10.1] Me [Q1–Q3]: 8.1 [6.9–9.7]	W = 19,858	—	<0.001	0.003

Y—yes; N—no; Me—median; Q1–Q3—interquartile range; W—Mann–Whitney U statistic reported by R; χ^2^—Pearson’s chi-square statistic; BH—Benjamini–Hochberg correction; FDR—false discovery rate. The eight *p*-values shown in this table were adjusted together as one exploratory family.

**Table 4:** Anatomical distribution of the largest index FLDIF per positive patient (*n* = 452).

Index-Finding Location	Number of Cases	Percentage
14–15	4	0.9%
24–25	3	0.7%
Region 26, peri-implant	1	0.2%
31–32	6	1.3%
33–34	3	0.7%
35–36	1	0.2%
36–37	7	1.5%
Left mandibular retromolar region (distal to 38)	209	46.2%
41–42	5	1.1%
43–44	2	0.4%
45–46	3	0.7%
46–47	5	1.1%
Right mandibular retromolar region (distal to 48)	203	44.9%

**Table 5:** Radiographic characteristics of the largest index FLDIF per positive patient (*n* = 452).

Parameter	Result
Largest index-finding diameter [mm]	Me [Q1–Q3]: 8.5 [7.1–9.8]
Minimum relative CBCT gray-scale value of index finding	Me [Q1–Q3]: −424.5 [−502.2 to −359.0]
Index finding visible on panoramic radiograph	180 (39.8%)
Index finding not visible on panoramic radiograph	272 (60.2%)

**Table 6:** Exploratory multivariable logistic regression for presence of the study-defined FLDIF phenotype (*n* = 1000).

Variable	OR	95% CI	*p*-Value
Age	1.015	1.006–1.025	0.001
Sex, male vs. female	0.927	0.719–1.194	0.556
Osteoporosis	0.743	0.292–1.893	0.529
Arterial hypertension	1.140	0.810–1.604	0.453
Cardiovascular disease	1.050	0.662–1.665	0.836
Diabetes mellitus	1.647	1.022–2.686	0.042

## Data Availability

The data supporting the findings of this retrospective observational study are available from the corresponding author upon reasonable request. Due to privacy and ethical restrictions, the data are not publicly available.

## Supplementary Materials

The following supporting information can be downloaded at: https://www.mdpi.com/article/10.3390/jcm15176636/s1, Supplementary Figure S1. Q–Q plot of age in the entire study cohort; Supplementary Figure S2. Q–Q plot of age in FLDIF-positive patients; Supplementary Figure S3. Q–Q plot of relative CBCT gray-scale values for index findings visible on panoramic radiography; Supplementary Figure S4. Q–Q plot of index-finding size for index findings visible on panoramic radiography; Supplementary Figure S5. Q–Q plot of largest index-finding diameter; Supplementary Figure S6. Q–Q plot of minimum relative CBCT gray-scale value; Supplementary Figure S7. Influential observations assessed using Cook’s distance; Supplementary Table S1. Multicollinearity assessment using variance inflation factors; Supplementary Table S2. Functional-form assessment, influential-observation sensitivity analysis, and model-fit statistics for the multivariable logistic regression; Supplementary Table S3. Summary of statistical functions and model specification used for analysis.
