# Supplementary material for: Frequency and Systemic Associations of Focal Low-Trabeculation/Low-Density Intramedullary Jawbone Findings: A Retrospective CBCT Study
Source: J Clin Med. 2026 Aug 27;15(17):6636. doi: 10.3390/jcm15176636 (PMC13566813; doi:10.3390/jcm15176636)
Supplement: Supplementary file 1 [file jcm-15-06636-s001.zip › jcm-4499196-supplementary.pdf]

Supplementary materials

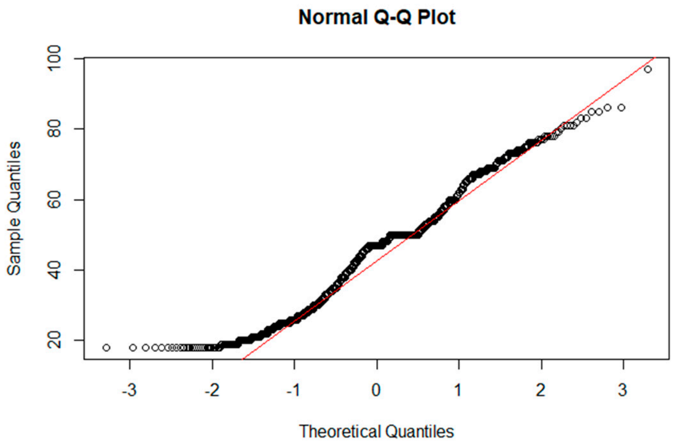

Supplementary Figure S1. Q–Q plot of age in the entire study cohort.

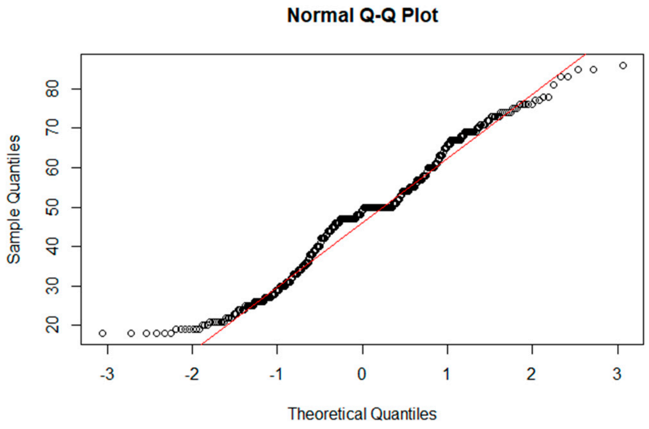

Supplementary Figure S2. Q–Q plot of age in FLDIF-positive patients.

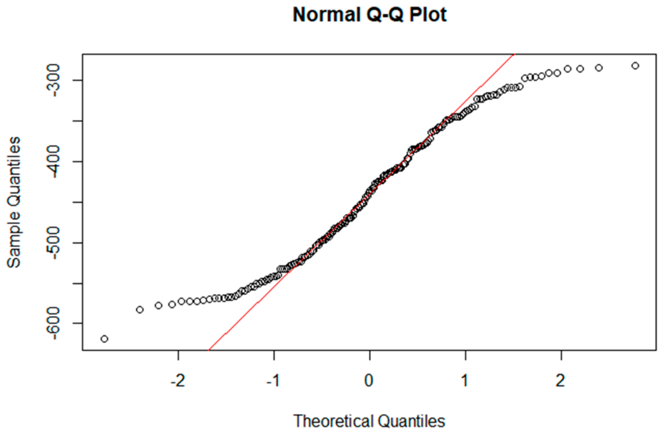

Supplementary Figure S3. Q–Q plot of relative CBCT gray-scale values for index findings visible on panoramic radiography.

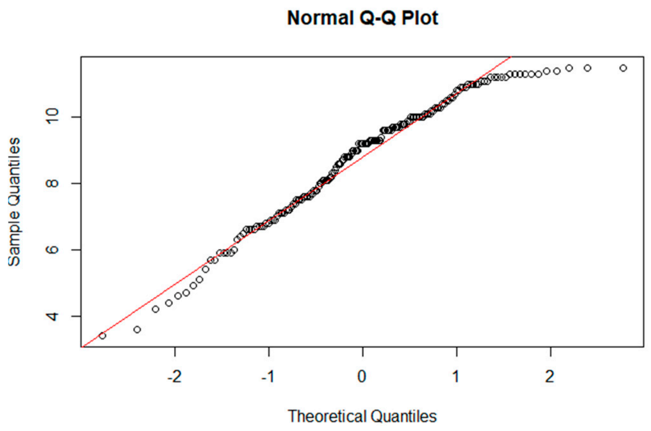

Supplementary Figure S4. Q–Q plot of index-finding size for index findings visible on panoramic radiography.

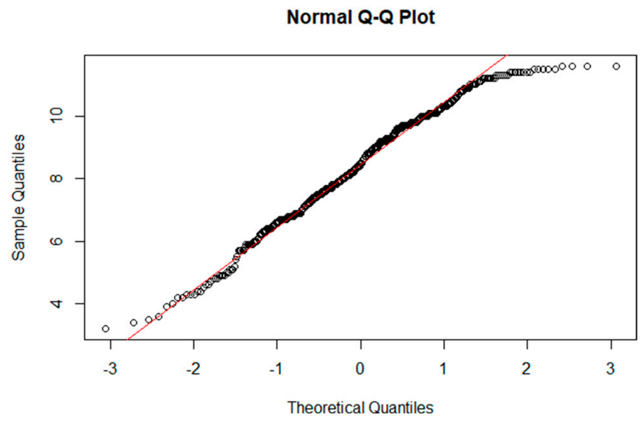

Supplementary Figure S5. Q–Q plot of largest index-finding diameter.

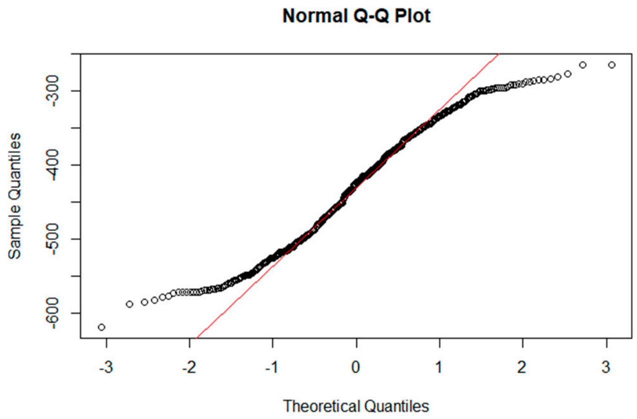

Supplementary Figure S6. Q–Q plot of minimum relative CBCT gray-scale value.

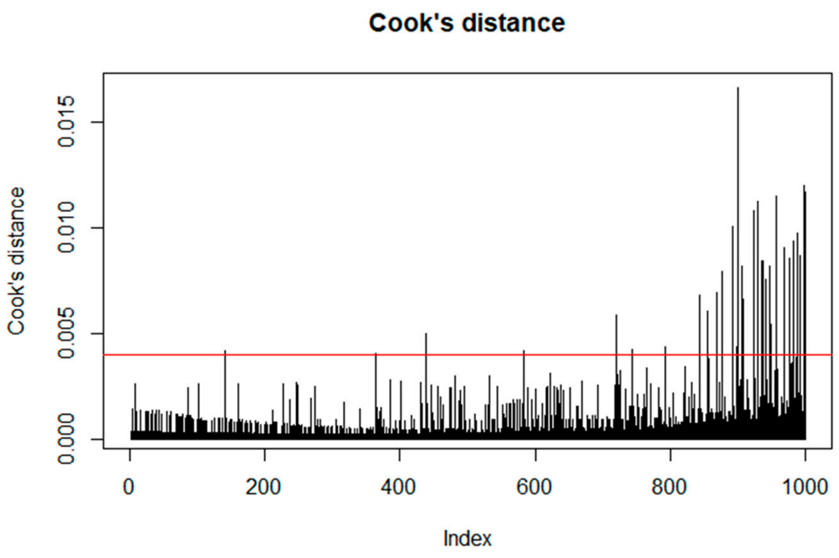

Supplementary Figure S7. Influential observations assessed using Cook's distance.

Supplementary Table S1. Multicollinearity assessment using variance inflation factors.

Variable	VIF
Age	1.39
Sex	1.01
Osteoporosis	1.08
Arterial hypertension	1.23
Cardiovascular disease	1.08
Diabetes mellitus	1.03

Supplementary Table S2. Functional-form assessment, influential-observation sensitivity analysis, and model-fit statistics for the multivariable logistic regression.

Diagnostic / sensitivity analysis	Result
Age×log(age) functional-form test	$p=0.41$
Restricted cubic-spline versus linear-age model	$p=0.37$
Observations above the 4/n Cook's-distance reference	7
Sensitivity model: age	OR 1.015 (95% CI 1.005–1.026); $p=0.004$
Sensitivity model: diabetes mellitus	OR 1.59 (95% CI 0.98–2.59); $p=0.061$
Likelihood-ratio test versus intercept-only model	$\chi^2=27.6$; df=6; $p<0.001$
Akaike information criterion	AIC=1368.2

Nagelkerke R^2	0.041
Hosmer–Lemeshow goodness-of-fit test	χ^2=7.1; df=8; p=0.53

Supplementary Table S3. Summary of statistical functions and model specification used for analysis.

Analysis purpose	R function / analysis specification
Data import	library(readxl); data <- read_excel(file.choose())
Median and quartiles	median(x, na.rm=TRUE); quantile(x, c(0.25, 0.75), na.rm=TRUE)
Counts and percentages	table(x); prop.table(table(x))*100
95% CI for a proportion	prop.test(x, n)
Q–Q plot	qqnorm(x); qqline(x, col="red")
Mann–Whitney U test	wilcox.test(y ~ group, data=...)
Pearson chi-square test	chisq.test(table(group, outcome))
FDR correction	p.adjust(p_values, method="BH")
Boxplot	boxplot(y ~ group, data=..., col="lightgray", medlwd=3)
Logistic regression	model <- glm(FLDIF_status ~ Age + Sex_bin + Osteoporosis_bin + Hypertension_bin + CVD_bin + Diabetes_bin, data=data, family=binomial)
OR and 95% CI	exp(cbind(OR=coef(model), confint(model)))
Variance inflation factor	library(car); vif(model)
Cook's distance	cooksD <- cooks.distance(model); plot(cooksD, type="h"); abline(h=4/nrow(data), col="red")
Age×log(age) functional-form assessment	glm(FLDIF_status ~ Age + I(Age*log(Age)) + Sex_bin + Osteoporosis_bin + Hypertension_bin + CVD_bin + Diabetes_bin, data=data, family=binomial)
Restricted cubic-spline comparison	Fit a restricted cubic-spline specification for age and compare it with the linear-age model using a likelihood-ratio test.
Influential-observation sensitivity model	Refit the same multivariable logistic model after excluding observations with Cook’s distance >4/n.

Overall model likelihood-ratio test	anova(null_model, model, test='LRT')
AIC	AIC(model)
Nagelkerke R^2	Calculate Nagelkerke R^2 for the fitted binomial logistic model.
Hosmer–Lemeshow goodness-of-fit	Perform a Hosmer–Lemeshow goodness-of-fit test using 10 risk groups.

Note: Variable names in Supplementary Table S3 are standardized descriptive labels used to summarize the analysis specification; they are not intended to reproduce the internal dataset column names verbatim.
